# Supplementary material for: GFP-on mouse model for interrogation of in vivo gene editing
Source: Nat Commun. 2025 Jul 31;16:7017. doi: 10.1038/s41467-025-61449-y (PMC12313916; doi:10.1038/s41467-025-61449-y)
Supplement: Supplementary file 1 — Supplementary Information [file 41467_2025_61449_MOESM1_ESM.pdf]

GFP-on mouse model for interrogation of *in vivo* gene editing

Supplementary Information

Supplementary Figure 1. Mutation selection for GFP-on mouse generation.

Supplementary Figure 2. Detailed assessment of GFP-on$^{pm/pm}$ reporter mouse model.

Supplementary Figure 3. CIRCLE-seq nomination of Cas-dependent ABE off-targets.

Supplementary Figure 4. Targeted amplicon sequencing of loci nominated by CIRCLE-seq.

Supplementary Figure 5. Detailed assessment of GFP-on$^{pm/pm}$ mice injected with AAV9-SpABE8e-sgRNA1.

Supplementary Figure 6. Whole body imaging of SCID GFP-on$^{pm/pm}$ mice injected with dual AAV9-SpABE8e-sgRNA1 or single AAV9-SaABE8e-EGFP^{Q81X}sgRNA using the cryo-macrotome.

Supplementary Figure 7. Quantitative analysis of volumetric cryo-macrotome mice

Supplementary Figure 8. Evaluation of EGFP *in utero* editing in GFP-on$^{-/pm}$

Supplementary Table 1. Oligonucleotides

Supplementary Table 2. FACS antibodies list

Supplementary Figure 1: Mutation selection for GFP-on mouse generation. Sequences of the candidate mutations at EGFP Q70X, Q81X, Q95X with CBE protospacer and protospacer adjacent motif (PAM) sequences underlined in purple. The introduced Q81X mutation has accessible nearby PAMs for SpABE8e, SauriABE8e, and SaABE8e (underlined) for which the target A falls within the active window of the respective ABE8e orthologue.

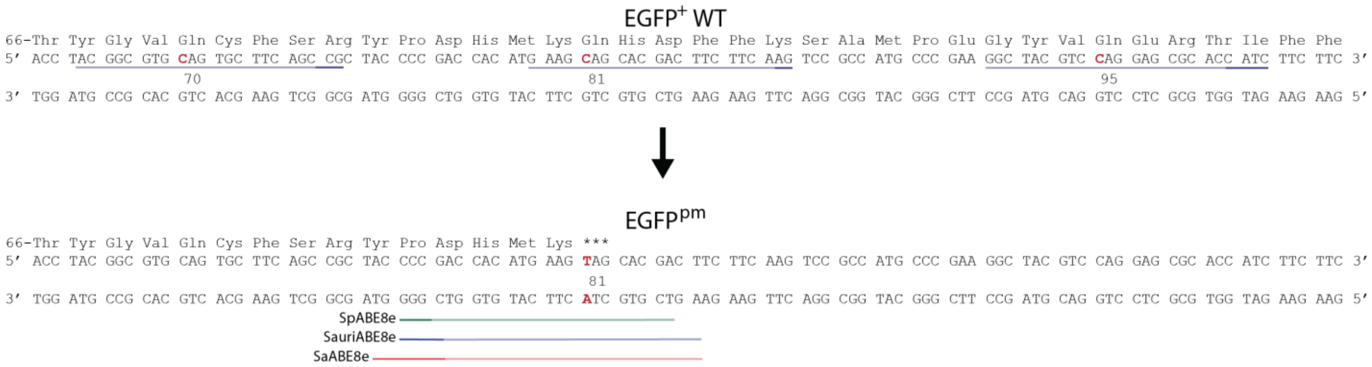

Supplementary Figure 2: Detailed assessment of GFP-on$^{pm/pm}$ reporter mouse model. Assessment of EGFP expression in the (a) peripheral blood and (b) bone marrow of GFP-on$^{pm/pm}$ by flow cytometry showing loss of EGFP expression in all lineages compared to EGFP^{+} mouse.

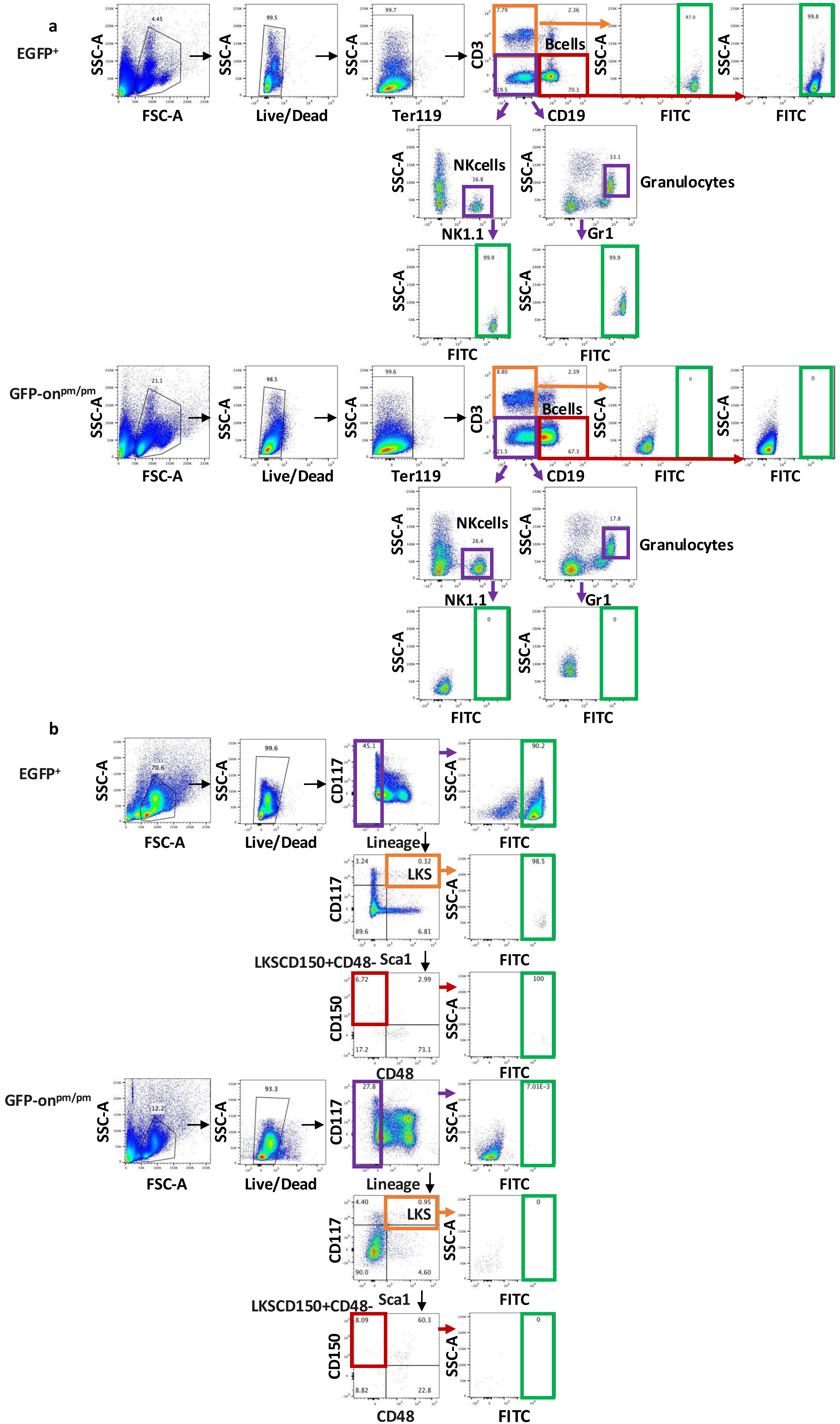

Supplementary Figure 3: CIRCLE-seq nomination of Cas-dependent ABE off-targets. Off-target sites aligned to the ABE sgRNA1 sequence which were evaluated for genomic off-target editing.

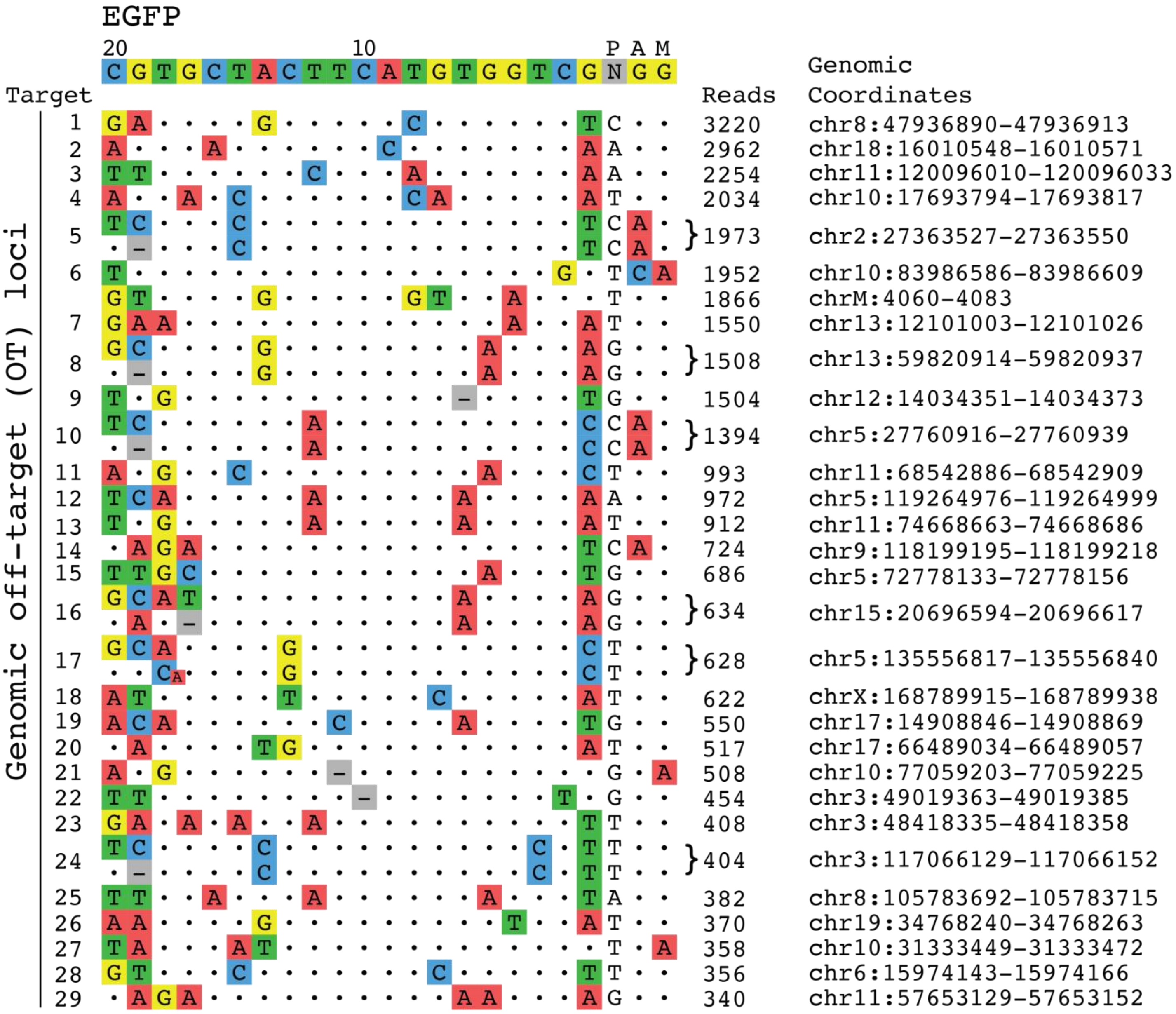

Supplementary Figure 4: Targeted amplicon sequencing of loci nominated by CIRCLE-seq. Off-target (OT) ABE editing efficiencies across top 29 off-target loci nominated by CIRCLE-seq for heart tissue from in vivo injected (a) EGFP$^{pm/pm}$ and (b) EGFP$^{pm/-}$ mice, and (c) ex vivo treated EGFP$^{pm/pm}$ derived fibroblasts. Unreported sites did not PCR amplify. No statistically significant sites were identified. Error bars represent standard deviation and bars represent the mean.

a

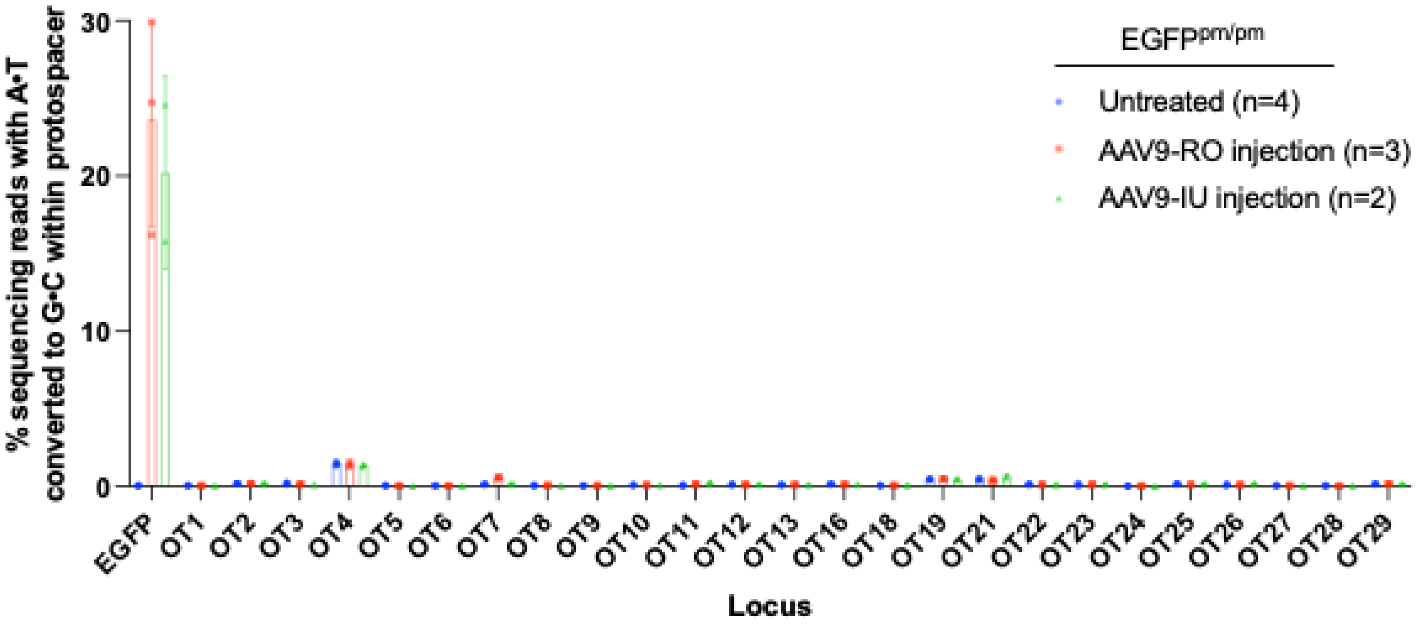

b

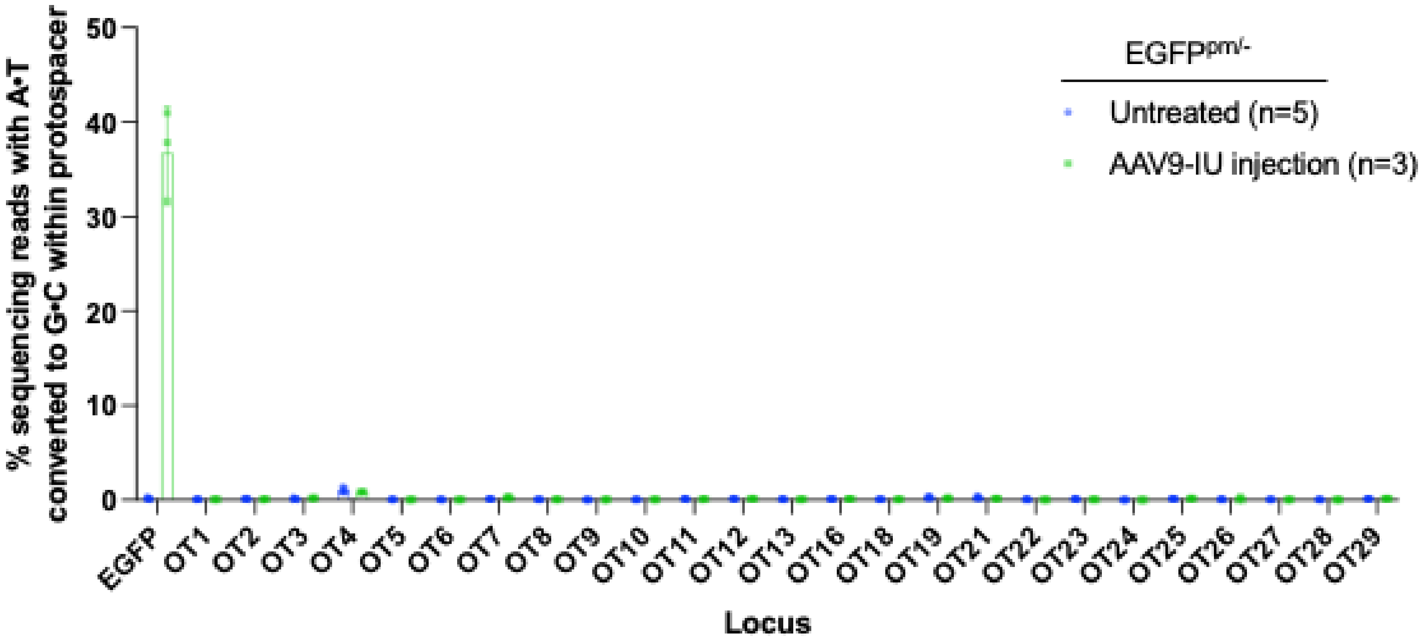

c

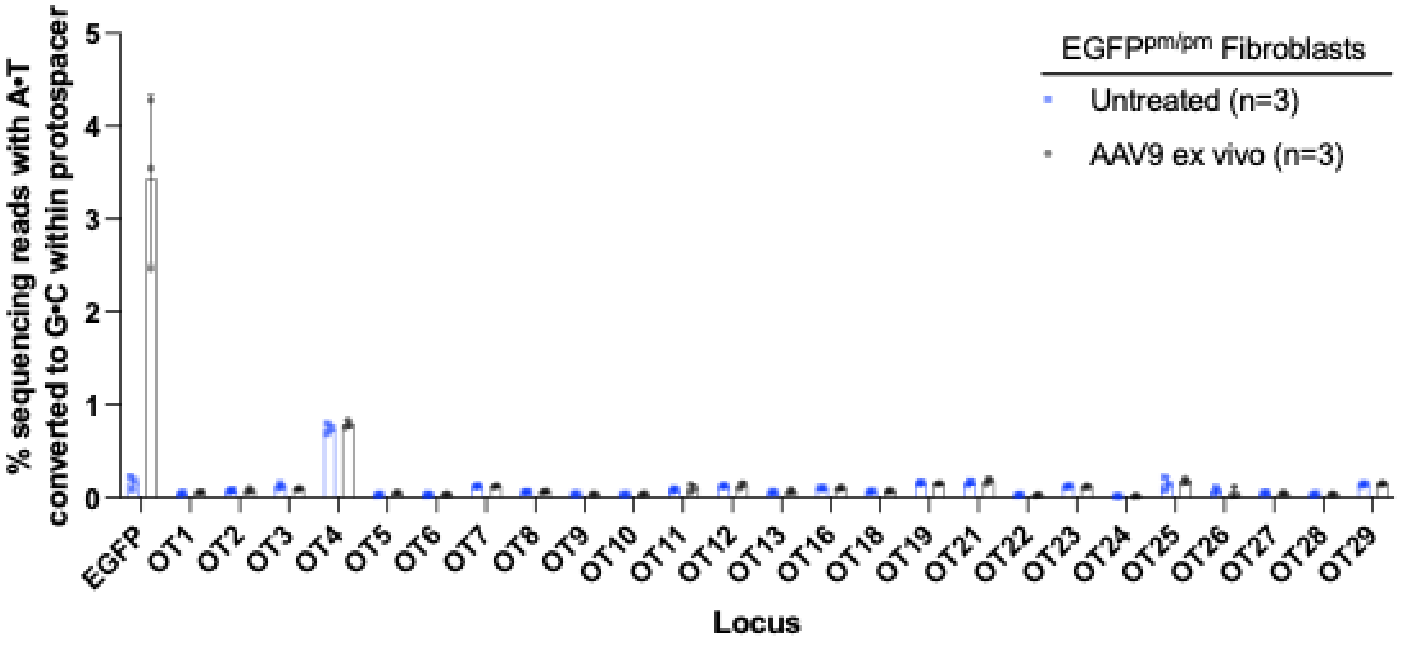

Supplementary Figure 5: Detailed assessment of GFP-on$^{pm/pm}$ mice injected with AAV9-SpABE8e-sgRNA1. (a) EGFP restoration in mice injected with dual AAV9-SpABE8e. (b) FACS plots showing EGFP expression in the bone marrow with distribution represented in Lineage-, CD117+Sca+ cells, and CD150+CD48- HSCs, as well as (c) EGFP expression in peripheral blood of AAV9-treated mice.

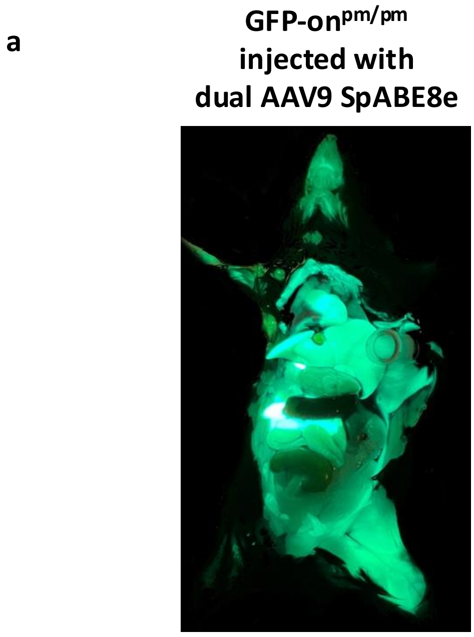

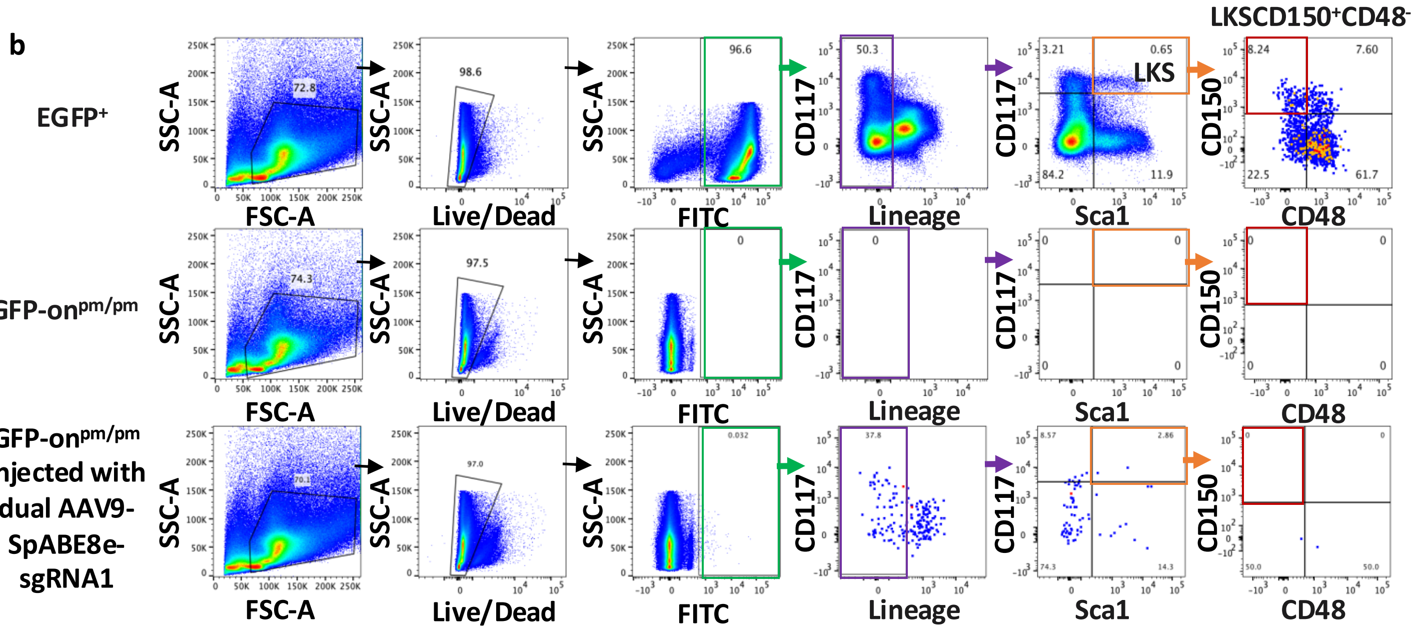

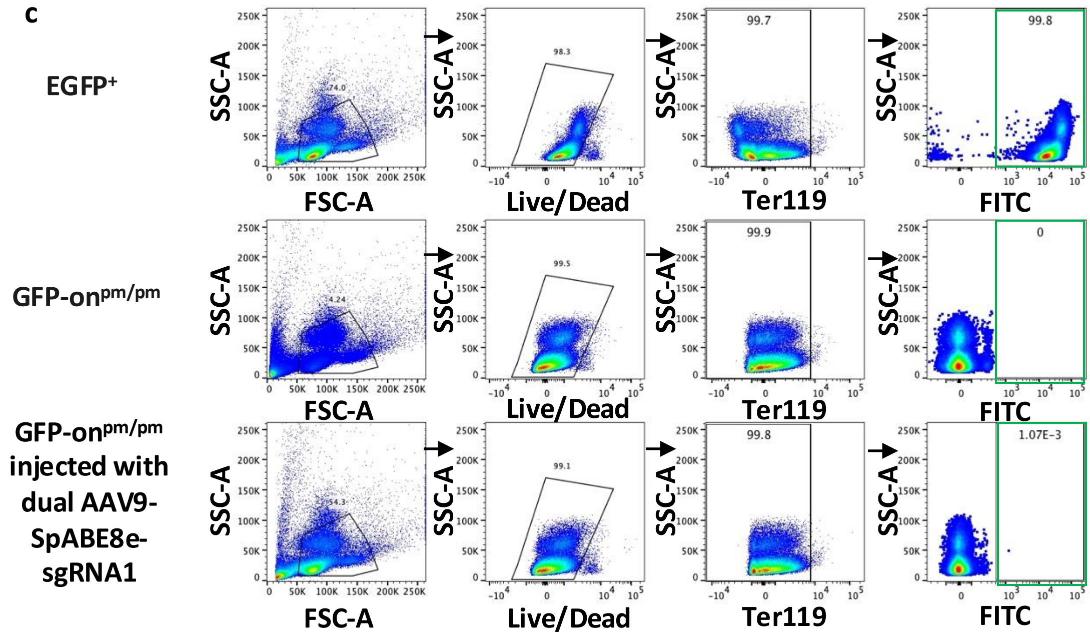

Supplementary Figure 6: Whole body imaging of SCID GFP-on$^{pm/pm}$ mice injected with dual AAV9-SpABE8e-sgRNA1 or single AAV9-SaABE8e-EGFP^{Q81X}sgRNA using the cryo-macrotome. (a) Whole body EGFP fluorescence for each mouse cryo-imaged, shown as maximum intensity projection (MIP) images. Corresponding RGB volumes and segmented organ surfaces for analysis are shown for each mouse. (b) A sampling of EGFP fluorescence image slices of each organ (between 1/10 and 1/5 sampling of slices).

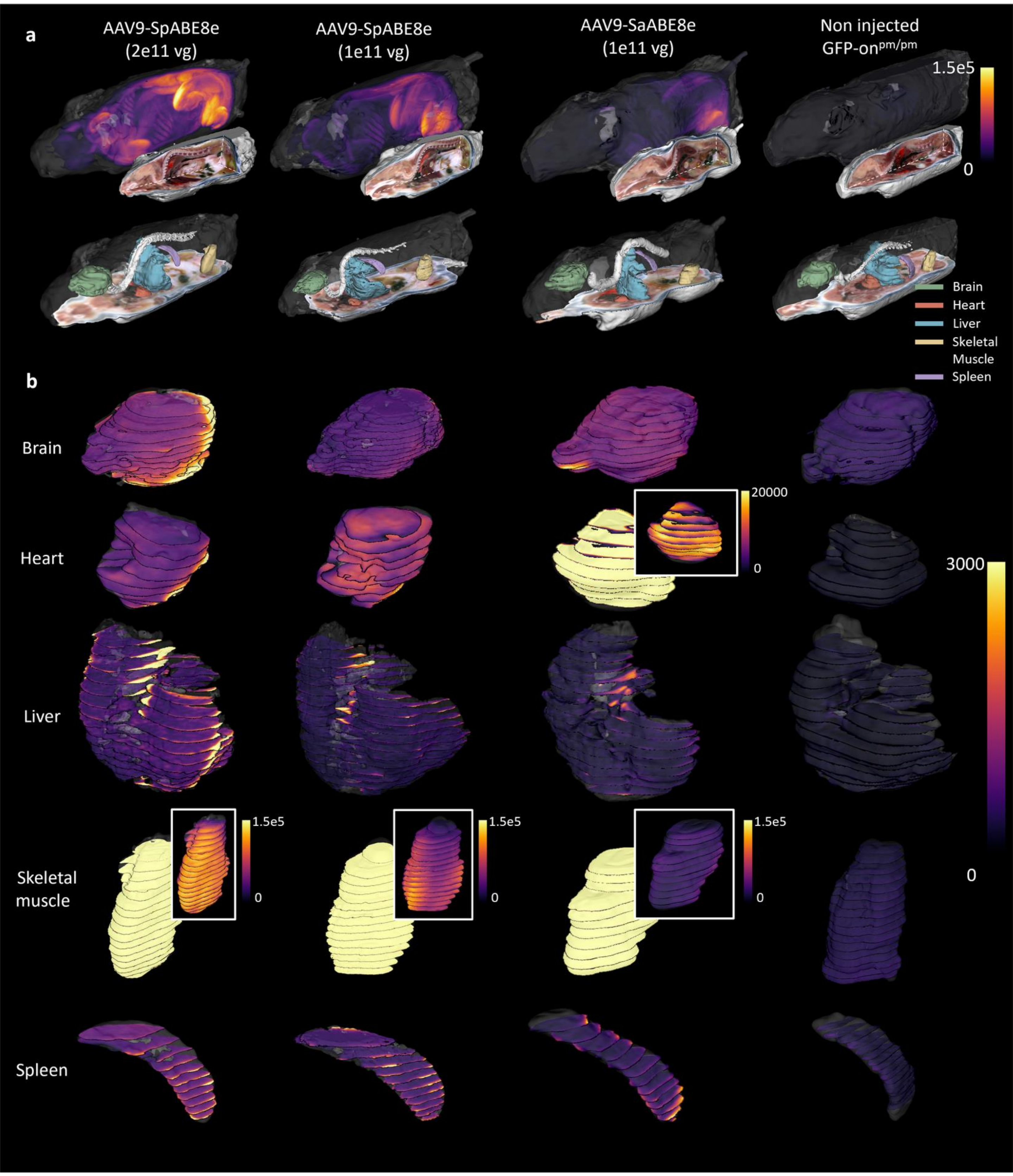

Supplementary Figure 7: Quantitative analysis of volumetric cryo-macrotome mice. Mean signal in each organ and the percent of organ volume above a 3 standard deviation threshold determined by the control mouse signal.

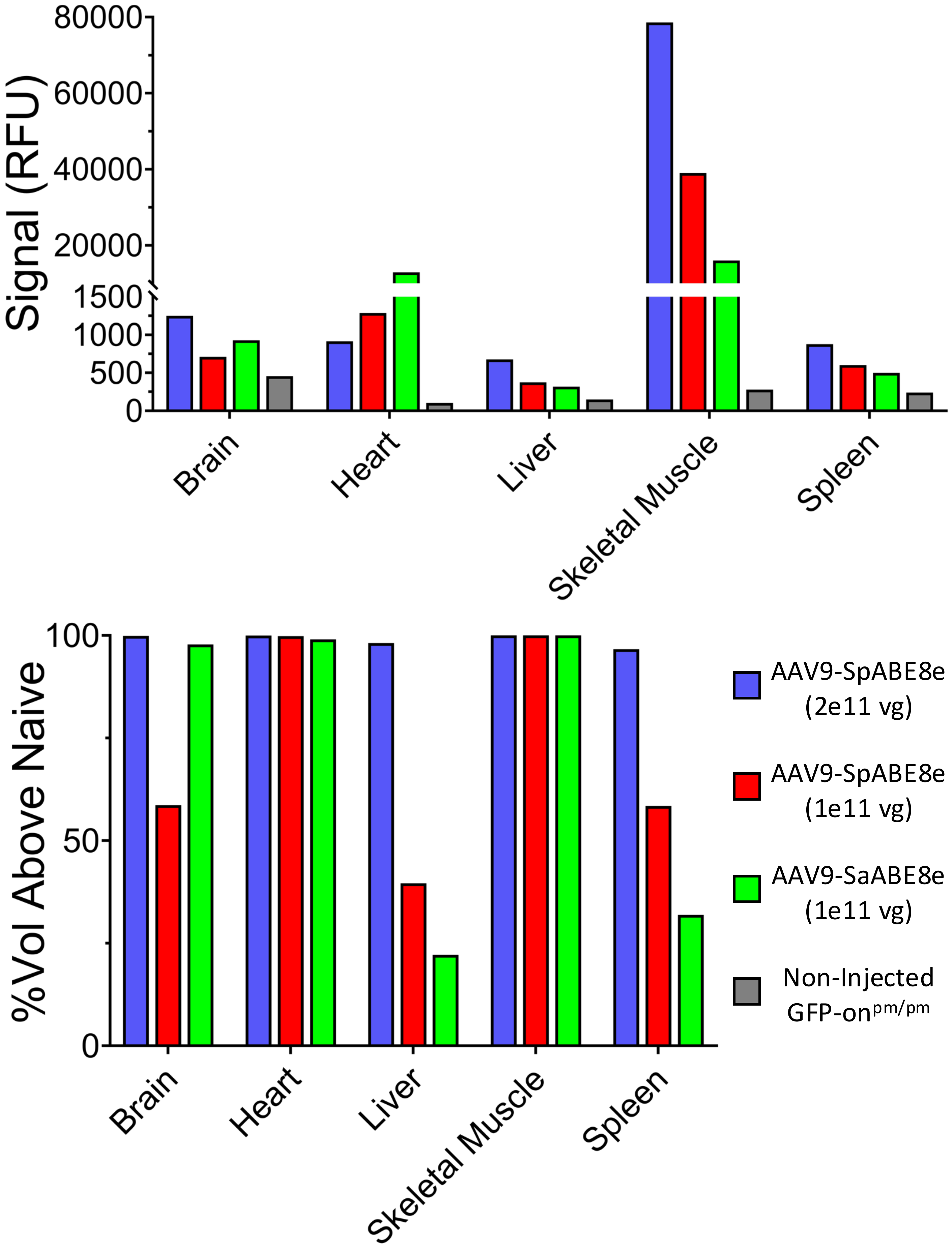

Supplementary Figure 8: Evaluation of EGFP *in utero* editing in GFP-on$^{-/pm}$. (a) EGFP Visualization in GFP-on$^{-/pm}$ pups treated *in utero* with dual AAV9-SpABE8e (n=4, biological replicates). (b) Flow cytometry, (c) immunofluorescence (10X magnification, 100um), (d) fluorescence microscopy (10X magnification, 100µm), and (e) HTS showing editing in various organs. Data are presented as mean. Abbreviations: PB, peripheral blood; BM, bone marrow.

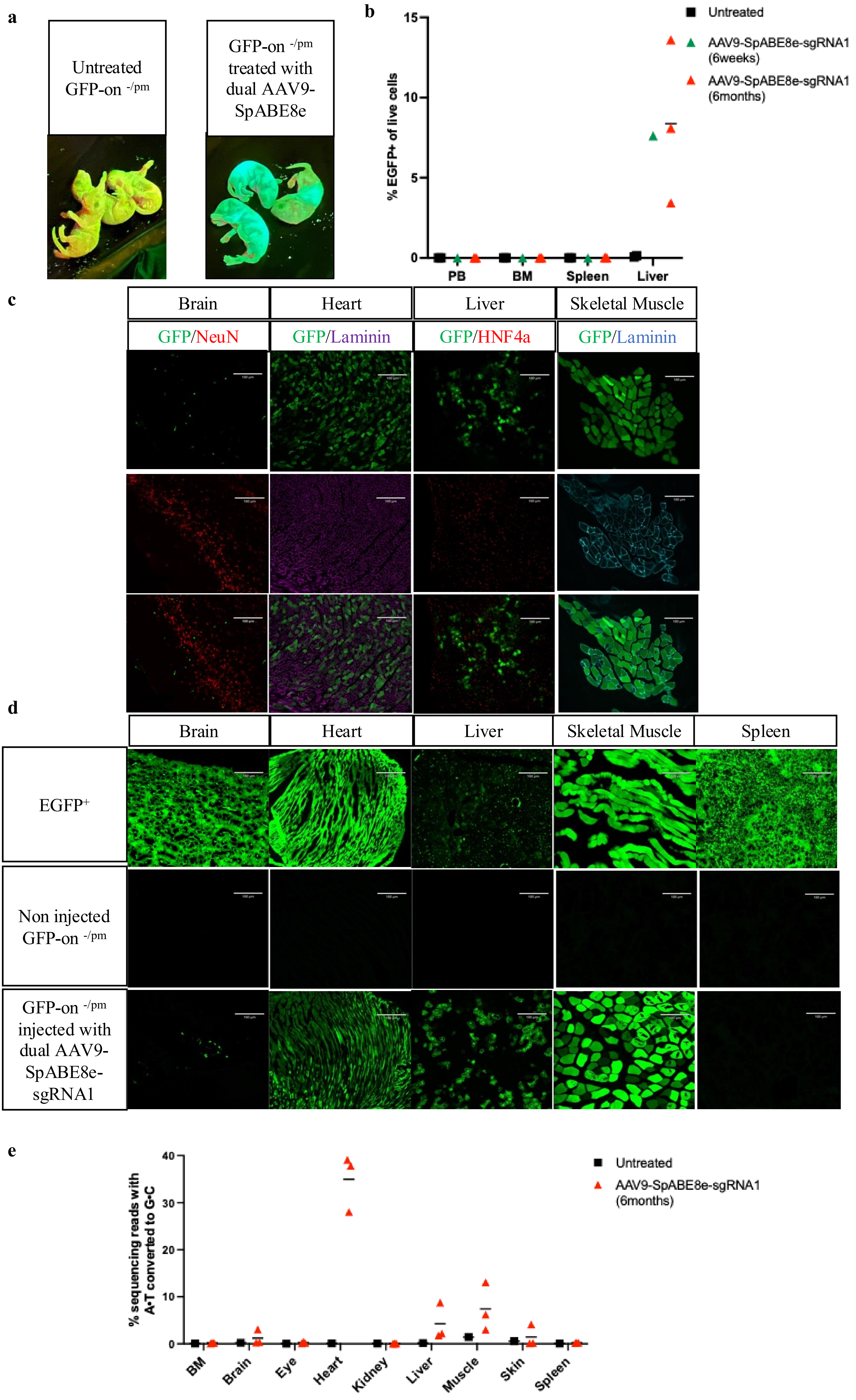

Supplementary Table 1: Oligonucleotides

Name	Sequence 5' to 3'
EGFP HTS_fwd	ACACTCTTTCCCTACACGACGCTCTTCCGATCTNNNNCCTTGAAGAAGATGGTGC
EGFP HTS_rev	TGGAGTTCAGACGTGTGCTCTTCCGATCTGCAACGTGCTGGTTATTGTG
OT1 HTS_fwd	ACACTCTTTCCCTACACGACGCTCTTCCGATCTNNNNAAAGTGCTGCTGAGACAGGACAA
OT1 HTS_rev	TGGAGTTCAGACGTGTGCTCTTCCGATCTAGATGGACCATGGATCCCAGTGA
OT2 HTS_fwd	ACACTCTTTCCCTACACGACGCTCTTCCGATCTNNNNCTCCCTTGAACAATGCTGTGATT
OT2 HTS_rev	TGGAGTTCAGACGTGTGCTCTTCCGATCTAGCACAGCAGGACTGGGGTTTA
OT3 HTS_fwd	ACACTCTTTCCCTACACGACGCTCTTCCGATCTNNNNTTGCTACCCACACAGCAGGC
OT3 HTS_rev	TGGAGTTCAGACGTGTGCTCTTCCGATCTTAAGGGCCGTGTTGCAGGTG
OT4 HTS_fwd	ACACTCTTTCCCTACACGACGCTCTTCCGATCTNNNNGGGACCATAAGAGGGTGTTCAGT
OT4 HTS_rev	TGGAGTTCAGACGTGTGCTCTTCCGATCTGGGCCTTCTGATGACAGGCTTT
OT5 HTS_fwd	ACACTCTTTCCCTACACGACGCTCTTCCGATCTNNNNGGGCAGCAGTAAGCACCCATAA
OT5 HTS_rev	TGGAGTTCAGACGTGTGCTCTTCCGATCTAGAGTATGGGGAGCCTCAAACCAG
OT6 HTS_fwd	ACACTCTTTCCCTACACGACGCTCTTCCGATCTNNNNAGAGCCCTGAAGCTCCTGCT
OT6 HTS_rev	TGGAGTTCAGACGTGTGCTCTTCCGATCTAGTGGTCACAGGGTAGGGGG
OT7 HTS_fwd	ACACTCTTTCCCTACACGACGCTCTTCCGATCTNNNNACAAAGTGAGCCCCAAAAGCAC
OT7 HTS_rev	TGGAGTTCAGACGTGTGCTCTTCCGATCTAGCCTCACTCCGCCAAATGT
OT8 HTS_fwd	ACACTCTTTCCCTACACGACGCTCTTCCGATCTNNNNTGTGCACATTTTGGAAAAATACA
OT8 HTS_rev	TGGAGTTCAGACGTGTGCTCTTCCGATCTAGGACCCCGAGTAGTTGGAA
OT9 HTS_fwd	ACACTCTTTCCCTACACGACGCTCTTCCGATCTNNNNGAGGGAGCCGATGGGGATTG
OT9 HTS_rev	TGGAGTTCAGACGTGTGCTCTTCCGATCTATCCCTCTTGCCTGGAGCAC
OT10 HTS_fwd	ACACTCTTTCCCTACACGACGCTCTTCCGATCTNNNNCACAATGACACCTGCCTCTTTAC
OT10 HTS_rev	TGGAGTTCAGACGTGTGCTCTTCCGATCTCCTGTCCAATCACAGGCTTCTT
OT11 HTS_fwd	ACACTCTTTCCCTACACGACGCTCTTCCGATCTNNNNAACATTGAGGCAGGCAGAGGATT
OT11 HTS_rev	TGGAGTTCAGACGTGTGCTCTTCCGATCTACCTTTGACACCAGCGTCAGG
OT12 HTS_fwd	ACACTCTTTCCCTACACGACGCTCTTCCGATCTNNNNCTGTGTTGGGGTATGCTAACTGG
OT12 HTS_rev	TGGAGTTCAGACGTGTGCTCTTCCGATCTATGTGCAGCAAGGATGGAGGAT
OT13 HTS_fwd	ACACTCTTTCCCTACACGACGCTCTTCCGATCTNNNNCCACCTGCCTCTGTCGTCTT
OT13 HTS_rev	TGGAGTTCAGACGTGTGCTCTTCCGATCTTGAGGCCAGCTCACTCCAAC
OT16 HTS_fwd	ACACTCTTTCCCTACACGACGCTCTTCCGATCTNNNNACTCAGCTTGACACCATGTTAGG
OT16 HTS_rev	TGGAGTTCAGACGTGTGCTCTTCCGATCTGCTTGAGCCTTGGTTTTGGCTTT
OT18 HTS_fwd	ACACTCTTTCCCTACACGACGCTCTTCCGATCTNNNNAGATCCTTGATAAGAGCATACCT
OT18 HTS_rev	TGGAGTTCAGACGTGTGCTCTTCCGATCTTGGCACCATGCTTGTTTGTGT
OT19 HTS_fwd	ACACTCTTTCCCTACACGACGCTCTTCCGATCTNNNNAAGCCCACTGGCCAACTGAC
OT19 HTS_rev	TGGAGTTCAGACGTGTGCTCTTCCGATCTGTGCTGGCTGTCACTGTCCC
OT21 HTS_fwd	ACACTCTTTCCCTACACGACGCTCTTCCGATCTNNNNCCTCCCCACCCCTACTCCAG
OT21 HTS_rev	TGGAGTTCAGACGTGTGCTCTTCCGATCTGACCTCCAGGACCTCCCTCC
OT22 HTS_fwd	ACACTCTTTCCCTACACGACGCTCTTCCGATCTNNNNTTGGGGGAGGGATTGAAGGC
OT22 HTS_rev	TGGAGTTCAGACGTGTGCTCTTCCGATCTTTTGGGGTACCCGCATGGAG
OT23 HTS_fwd	ACACTCTTTCCCTACACGACGCTCTTCCGATCTNNNNTGGGTTTTTCGGAGGGCAGT
OT23 HTS_rev	TGGAGTTCAGACGTGTGCTCTTCCGATCTTGTGGTTCAGATGTTAGCTCTCAG
OT24 HTS_fwd	ACACTCTTTCCCTACACGACGCTCTTCCGATCTNNNNTGCCTTAAACAAAGTGATGTGGT
OT24 HTS_rev	TGGAGTTCAGACGTGTGCTCTTCCGATCTTCACTGTGTTGACGTGTGTGTCA
OT25 HTS_fwd	ACACTCTTTCCCTACACGACGCTCTTCCGATCTNNNNGGTCTGGTCCACCTGAAGAACCC
OT25 HTS_rev	TGGAGTTCAGACGTGTGCTCTTCCGATCTGGTGCCATTGTGAGGCAGAGTT
OT26 HTS_fwd	ACACTCTTTCCCTACACGACGCTCTTCCGATCTNNNNACAAAGGGAACATGGAAACGTGG
OT26 HTS_rev	TGGAGTTCAGACGTGTGCTCTTCCGATCTACTCCCACCCCACGCTTAGTG
OT27 HTS_fwd	ACACTCTTTCCCTACACGACGCTCTTCCGATCTNNNNAAGACATAACTAGAAGCCAATCC
OT27 HTS_rev	TGGAGTTCAGACGTGTGCTCTTCCGATCTGGCATGGTACCTGAGGCAAA
OT28 HTS_fwd	ACACTCTTTCCCTACACGACGCTCTTCCGATCTNNNNACAGCAGTAAATGTGAGCCATCC
OT28 HTS_rev	TGGAGTTCAGACGTGTGCTCTTCCGATCTACTCCAGTTTTGAAGCTGCCGA
OT29 HTS_fwd	ACACTCTTTCCCTACACGACGCTCTTCCGATCTNNNNGGGTACCCGCTCTTCTTGGC
OT29 HTS_rev	TGGAGTTCAGACGTGTGCTCTTCCGATCTTCCCAGGCTTGGGGTTCTCA

Supplementary Table 2: FACS antibodies list

Antibody	Antibody clone	Identifier	Supplier
Ter119	TER-119	Cat# 116220	Biolegend
CD3	17A2	Cat# 100246	Biolegend
CD19	MB19-1	Cat# 11-0191-85	Thermo Fisher Scientific
Gr1	RB6-8C5	Cat# 108424	Biolegend
Nk1.1	PK136	Cat# 108716	Biolegend
Lineage	17A2, RB6-8C5, RA3-6B2, Ter-119, M1/70	Cat# 133313	Biolegend
CD117	2B8	Cat# 105808	Biolegend
Sca1	D7	Cat# 108112	Biolegend
CD48	HM48-1	Cat# 103432	Biolegend
CD150	TC15-12F12.2	Cat# 115914	Biolegend
